# Effects of acute lysergic acid diethylamide on intermittent ethanol and sucrose drinking and intracranial self-stimulation in C57BL/6 mice

**DOI:** 10.1177/02698811221104641

**Published:** 2022-06-13

**Authors:** Lauri V Elsilä, Juliana Harkki, Emma Enberg, Alvar Martti, Anni-Maija Linden, Esa R Korpi

**Affiliations:** Department of Pharmacology, Faculty of Medicine, University of Helsinki, Helsinki, Finland

**Keywords:** Reward, intracranial self-stimulation, ethanol, lysergic acid diethylamide, sucrose

## Abstract

**Background:**

Psychedelics, like lysergic acid diethylamide (LSD), are again being studied as potential therapies for many neuropsychiatric disorders, including addictions. At the same time, the acute effects of psychedelics on rewarding behaviours have been scarcely studied.

**Aims:**

The current study aimed to clarify if LSD decreases binge-like ethanol drinking in mice, and whether the observed acute effects on ethanol consumption are generalizable to a natural reinforcer, sucrose, and if the effects resulted from aversive or reward-attenuating effects caused by LSD.

**Methods:**

The effects of acute LSD were examined using 2-bottle choice intermittent ethanol (20%) and sucrose drinking (10%), discrete-trial current-intensity threshold method of intracranial self-stimulation and short-term feeding behaviour assay in C57BL/6 male mice.

**Results:**

The results showed that acute 0.1 mg/kg, but not 0.05 mg/kg, dose (i.p.) of LSD reduced 2-h intermittent ethanol drinking transiently without any prolonged effects. No effects were seen in intermittent 2-h sucrose drinking. The tested LSD doses had neither effect on the intracranial self-stimulation current-intensity thresholds, nor did LSD affect the threshold-lowering, or rewarding, effects of simultaneous amphetamine treatment. Furthermore, LSD had small, acute diminishing effects on 2-h food and water intake.

**Conclusions:**

Based on these results, LSD decreases binge-like ethanol drinking in mice, but only acutely. This effect is not likely to stem from reward-attenuating effects but could be in part due to reduced consummatory behaviour.

## Introduction

Alcohol use disorder is a complex, chronically relapsing psychiatric disorder characterized by compulsive alcohol use despite harmful consequences (American Psychiatric Association, 2013). It is estimated to cause more than 3 million deaths worldwide annually (World Health Organization, 2018). Like addictive disorders in general, alcohol use disorder is commonly considered to be associated with dysfunctions of motivation and reward processing (Koob and Volkow, 2016). A common finding is that intoxicating doses of alcohol or other drugs of abuse causes a rapid release of dopamine into the *nucleus accumbens*, which in turn is associated with subjective increases of hedonic tone and feelings of pleasure, or the so-called high (Koob and Volkow, 2016). The seeking of this rewarding aspect is usually considered to be the starting point of drug taking, also known as the binge/intoxication phase (Koob and Volkow, 2016), which then in some people turns into a compulsive form. The proposed mechanisms behind the transition to compulsive behaviour include formation of maladaptive habits (Lüscher et al., 2020), increase in negative affect (dysphoria and anxiety) and concurrent excessive goal-directed decision-making (Hogarth, 2020). The rewarding phase, or incentive salience, is also considered – together with negative emotionality, executive functions and social environment – as a major factor that affects the risk to and treatment efficacy in substance use disorders (Witkiewitz et al., 2019) and, therefore, could be considered as a potential domain for treatment targeting.

The current pharmacotherapies approved for treating alcohol use disorder have highly variable clinical effectiveness, and apart from opioid antagonists, such as naltrexone, their efficacy, especially in reducing binge drinking, is not satisfactory (Kranzler and Soyka, 2018). The search for more efficient pharmacotherapies to reduce alcohol intake has led scientists to look into psychedelics (Bogenschutz, 2013). Interest in psychedelics, compounds with mixed serotonin receptor agonist actions, such as psilocybin or lysergic acid diethylamide (LSD) as potential therapeutics for psychiatric disorders, has re-emerged during the last decade (Carhart-Harris and Goodwin, 2017), but the idea of using them to treat problematic alcohol use has its roots in 1950s (Dyck, 2006). A recent meta-analysis investigated these earlier clinical studies and, based on the results of six randomized studies with more than 500 participants, reported that a single dose of LSD is associated with reductions in alcohol misuse lasting even up to 12 months (Krebs and Johansen, 2012). A more recent small proof-of-concept trial with psilocybin also had similar results, showing reductions in both amount of alcohol drinking and in the number of heavy drinking days (Bogenschutz et al., 2015).

Conversely, investigations on psychedelics using rodent models of ethanol intake have yielded mixed results. In a rat model of ethanol relapse drinking, repeated administration of moderate psilocybin doses decreased ethanol intake when ethanol was re-introduced after abstinence, but the investigators did not observe any long-lasting effects with either LSD or psilocybin (Meinhardt et al., 2020). On the other hand, Alper et al. (2018) showed a single dose of LSD in mice to decrease ethanol consumption using a 24-h two-bottle drinking model with the reductions lasting for more than 40 days. The drinking schedule used in this study led into a chronic and stable ethanol drinking behaviour. Intermittent schedules of ethanol availability, leading into more binge-like drinking behaviours, have not been studied with classical psychedelics, but the hallucinogenic serotonin 5-HT_2A/2C_ receptor agonist, 2,5-dimethoxy-4-iodoamphetamine (DOI), has been investigated with this alcohol schedule along the years and found to reduce intermittent ethanol consumption, both in mice (Oppong-Damoah et al., 2019) and in Long-Evans (Berquist and Fantegrossi, 2021) and heavy-drinking AA rats (Maurel et al., 1999, 2000).

Importantly, the acute effects of LSD on reward-linked behaviours in rodents have been scarcely studied (Marczynski and Burns, 1976). It is, therefore, possible that LSD could modify reward processing or the acute feelings of reward, and that it could cause prolonged changes in reward taking behaviours. This can be studied in an alcohol self-administration design, a common animal model of the binge/intoxication phase of addiction (Koob et al., 2014). In the present study, we originally aimed to study whether a single administration of LSD could reduce intermittent, binge-like ethanol drinking in mice transiently or in a prolonged fashion. As we observed acute but no long-term effects, we then went on to further elucidate if these acute effects could be seen with a natural reinforcer sucrose, and if LSD modulates intracranial self-stimulation (ICSS) behaviours, either alone or in combination with a known positive reinforcer d-amphetamine. Lastly, an experiment was carried out to study the effects of acute LSD treatment on homeostatic eating and drinking behaviours.

## Materials and methods

### Subjects and handling

Total of 109 C57BL/6JRj male mice (Janvier Labs, Le Genest-Saint-Isle, France) were used in this study.

The mice for the ethanol and sucrose drinking, and the food consumption studies were single-housed in individually ventilated cages (GR900, Tecniplast, Buguggiate, Italy) in reversed 12-h light cycle (lights off at 6 am). The mice tested in the ICSS were initially housed in pairs, but single-housed after the surgery (GR500, Tecniplast) with regular 12-h light cycle (lights off at 6 pm). The cages were provided with aspen bedding, nesting material, a plastic in-cage house and/or tube and a piece of wood. Basic rodent chow (Teklad, Envigo, Huntingdon, UK) and tap water were freely available. The animal tests were approved by the Animal Experiment Board in Finland (permission no. ESAVI/1172/04.10.07/2018) and conducted in accordance with the national and EU-level ethical and procedural guidelines.

In order to slowly accustom the mice to the experimenter’s handling, all mice went through a 5-day habituation routine before the start of the behavioural experiment, as described before (Elsilä et al., 2020). Shortly, the mice were exposed to gradually intensifying handling, ranging from the experimenter slowly moving a hand in the cage and only slightly touching the mice to lifting the mice away from the cage with an open palm and letting them freely explore the length of the experimenter’s arm. The mice were also accustomed to the grip needed for the intraperitoneal (i.p.) injections. The mice in the ICSS experiment were also habituated to being handled with a towel, which was used to wrap the animals when connecting the stimulator cable to the intracranial electrode. The procedure was performed systematically and resulted in the same minimum level of habituation with all mice prior to starting the experiment.

### Drugs, randomization and blinding

LSD (Sigma-Aldrich, St. Louis, MO, USA) and d-amphetamine sulphate (Dexedrine; Smith Kline & French Laboratories Ltd, London, UK) were both freshly dissolved in sterile saline on the day of the treatment. The dose of d-amphetamine was calculated as a free base. The LSD doses were chosen based on their ability to induce head twitch responses in mice, a behavioural proxy for hallucinogenic potency of psychedelic drugs: the starting point was 0.05 mg/kg, which is both approximately the proposed ED_50_ dose for head twitch responses in mice (Corne and Pickering, 1967; Halberstadt and Geyer, 2013) and the dose that has previously been shown to cause decreases in ethanol intake in mice (Alper et al. 2018); the higher doses were twofold increments (0.1 and 0.2 mg/kg) and included the approximate peak effect doses of head twitch responses in mice (Halberstadt and Geyer 2013). Systematic observation of head twitch responses during the experiments was not possible with the designs used, but since the responses with the doses from 0.1 mg/kg onwards are described in the literature (Halberstadt and Geyer, 2013) and have been quantified in the lab as part of our previous experiments (Elsilä et al., 2020), no separate verification test was run. The d-amphetamine dose was chosen based on the observed effects on ICSS in the literature (Elmer et al., 2010), and preliminary experiments in our lab (Lainiola and Linden, unpublished data). The timing of the treatments relative to the behaviours of interest was chosen so that the peak in the head twitch responses, around 5–10 min, would take place during the experiment (Halberstadt et al., 2020; Halberstadt and Geyer, 2013). Injection volume of 10 ml/kg i.p. was used.

The mice were assigned to the treatment groups with complete randomization, except for the ICSS experiment, where a Latin square-like method was used to balance the crossover treatment schedule. The investigators conducting the experiments and doing the initial data analysis were blind to the group assignments.

### Intermittent drinking

#### Ethanol

The mice (*n* = 30, aged 8 weeks) were habituated for the reversed light cycle, single housing and two-bottle setup for a week after arrival. During this week, the mice were also habituated to handling as described above.

**Figure 1.**
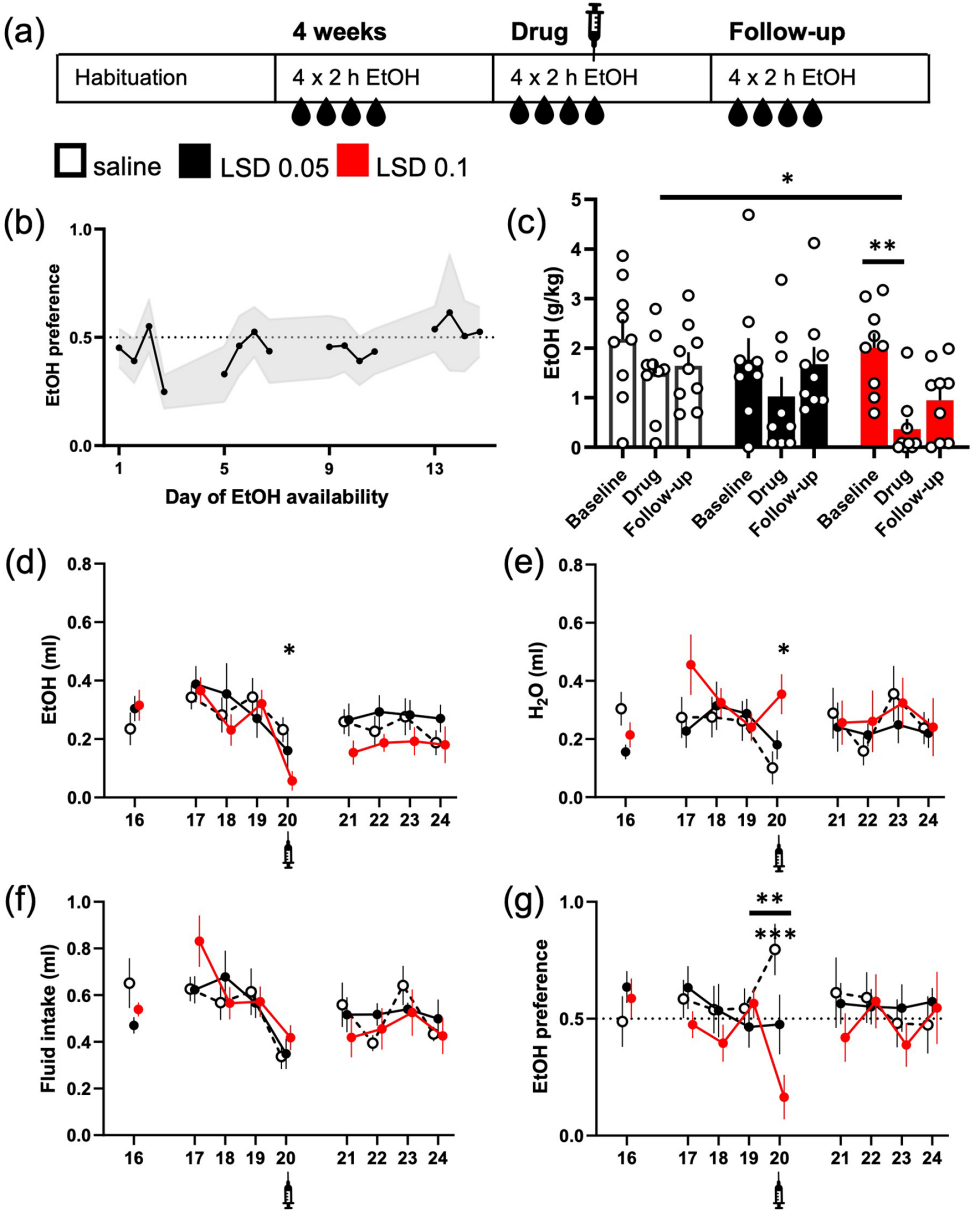
LSD acutely reduced intermittent ethanol intake. The design of the study is depicted in the diagram (a). The mice were given access to 20% (v/v) ethanol solution in their home cage for 2 h on four consecutive days. The mean ethanol intake increased during the weeks so that the mean preference of 50% was exceeded on the week 4 (b; data shown mean ± 95% confidence interval (CI)). Acute treatment with 0.05 or 0.1 mg/kg LSD (i.p.) decreased the ethanol consumption (g/kg, c; ml, d) compared to the saline control, but only the difference between the control and the dose of 0.1 mg/kg reached statistical significance. The intake was significantly lower also compared to the treatment group’s own baseline. While the larger dose of LSD also significantly increased water intake on the day of treatment (e, day 20), the total fluid intake was slightly reduced in all groups (f). Only the larger dose of LSD significantly decreased ethanol preference on the day of treatment compared to the day before (g, **), whereas the changes in the other two groups were non-significant. The difference between the saline control group and the 0.1 mg/kg LSD treatment group in the preference on the day of treatment was highly significant (g, ***). The numbers on the *X*-axis show the days when ethanol was available. Syringe symbols mark the injections. Data shown as mean ± SEM, circles in (c) show individual data points. SEM: standard error of the mean. *p < 0.05, **p < 0.01, ***p < 0.001.

To initiate ethanol drinking, the mice were introduced to 20% (v/v) ethanol in a plastic pipette with a stainless-steel double-ball bearing drinking tube 3 h after the beginning of the dark phase. The ethanol was available for 2 h on four consecutive days per week (Figure 1(a)). Water was always available in a regular drinking bottle, and the placement of the ethanol tube was varied to avoid place preferences. The ethanol intake was measured by volume, water intake by weighing. An identical, empty cage was used to control for spills and evaporation of both liquids.

After 4 weeks of drinking, the mice (*n* = 27) were randomized into three treatment groups. Three mice were excluded from the experiment due to non-existent ethanol intake (see Supplemental Table S1). On the day 4 of the week 5, the mice were treated with saline or LSD (0.05 or 0.1 mg/kg i.p.; *n* = 9 for each group) and immediately given access to ethanol. The ingested volumes were measured after 2 h. The original drinking pattern was continued for the following week to observe any possible long-term changes in the drinking behaviour.

#### Sucrose

**Figure 2.**
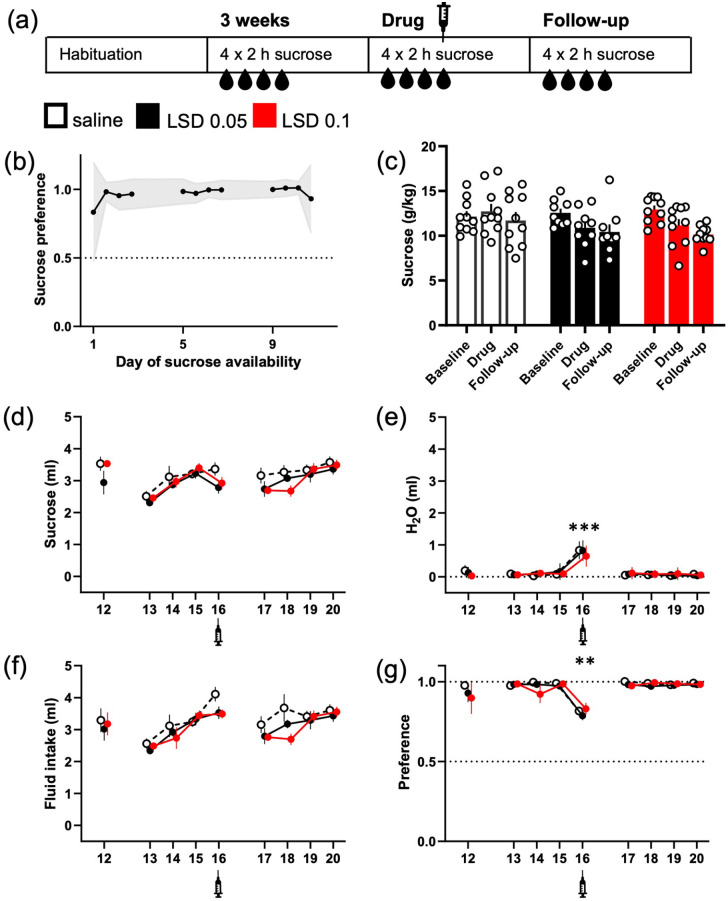
LSD did not significantly reduce intermittent sucrose consumption, but the water intake was increased in all treatment groups. The design of the study was mimicking the one used in the ethanol experiment, here depicted in the diagram (a). The mice were given access to 10% (w/v) sucrose solution in their home cage for 2 h on four consecutive days. The mice preferred the sucrose over water from the day 1 (b; data shown mean ± 95% CI). While a small decrease in the sucrose intake was observed in both LSD groups (g/kg, c; ml, d), no difference between the treatment groups or within the groups was statistically significant. Water intake on the other hand was significantly increased in all three groups after the injections (e, day 16). This increase levelled up the small decreases seen in sucrose consumption when measuring the total fluid intake (f). The increase in water intake was also large enough to significantly decrease the sucrose preference regardless the treatment (g). The numbers on the *X*-axis show the days when the sucrose was available. Syringe symbols mark the injections. Data shown as mean ± SEM, circles in (c) show individual data points. **p < 0.01, ***p < 0.001.

The intermittent sucrose drinking experiment was conducted with identical design as the ethanol drinking described above. Shortly, after the habituation, the mice (*n* = 30, aged 8 weeks on arrival) were introduced to 10% (w/v) sucrose solution 3 h into the dark phase on four consecutive days per week. The ingested volumes of sucrose and water were measured after 2 h (Figure 2(a)).

On the week 4, the mice (*n* = 29, see Supplemental Table S1 for exclusion details) were randomly assigned to the three treatment groups, saline (*n* = 10) or LSD (0.05 mg/kg or 0.1 mg/kg i.p.; *n* = 9 and 10, respectively). The treatments were administered immediately before the sucrose was made available on the day 4 of the week. The drinking behaviour was again followed for a week.

**Figure 3.**
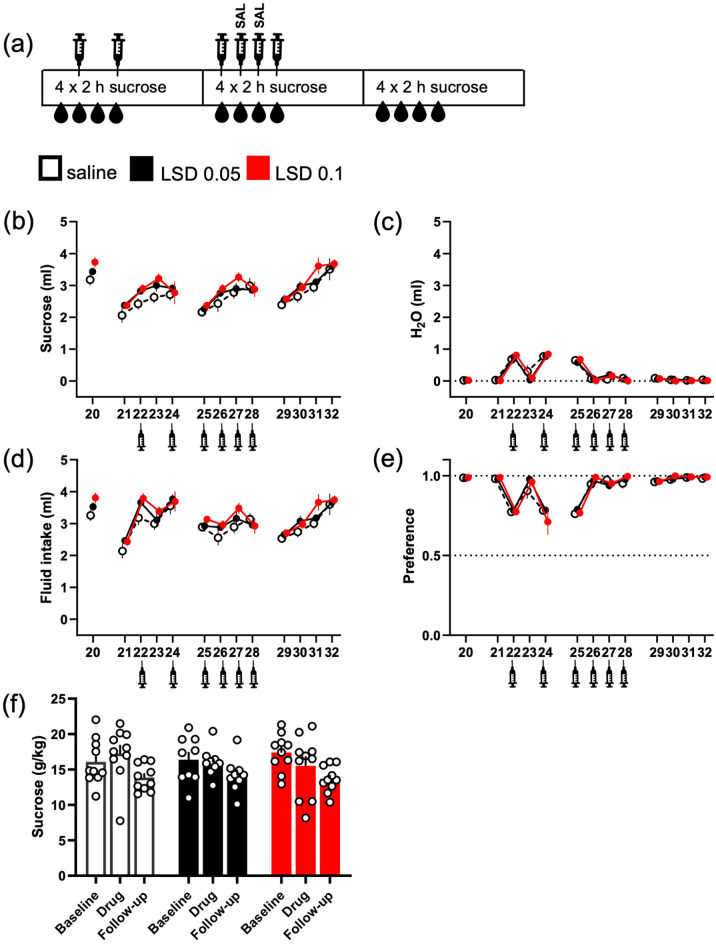
No effects of LSD on sucrose intake after repeated saline injections, which diminished the effect of injections on water intake. The original intermittent sucrose drinking experiment was continued with the same drinking schedule, but with added saline injections, as depicted in the diagram (a). When saline was injected every other day, the water intake increased on the day of injection and levelled back down to the baseline on the days without any addition (c). Repeated, daily injections before starting the 2-h measurement period diminished the effect, both on the water intake and the sucrose preference (e). Following this with another set of acute LSD treatments, again neither of the used doses significantly affected the sucrose drinking (ml, b; g/kg, f), the total fluid intake (d) or the sucrose preference (e). The numbers on the *X*-axis show the days when sucrose was available. Syringe symbols mark the injections. Data shown as mean ± SEM, circles in (f) show individual data points.

As described in more detail later in Section ‘Results’, the injections themselves, including saline injections, caused significant effects on water intake (Figure 2(e) and (g)). To control for these effects, the experiment was repeated with the same mice. For 2 weeks, all mice received saline injections before the sucrose was made available mimicking the drug treatment situation and making the mice more habituated to being injected; see Figure 3(a) for detailed description of the injection timings. On the day 4 of the week 2, the mice were again randomly assigned to the drug treatment groups (saline *n* = 10; LSD 0.05 *n* = 9; LSD 0.1 *n* = 10), and the above-mentioned treatment schedule was repeated. The drinking behaviour was monitored for a week after the treatment.

### Eating and drinking behaviours

The single-housed mice (*n* = 29, aged 18 weeks) had regular chow available, and water in a single bottle. For the baseline assessment of eating and drinking, the food pellets on the metal grid tray and the water bottles were weighed 3 h after the start of the dark phase. The weighing was then repeated at 2-h timepoint. The same measurements were repeated the following day for a second baseline data point. The timing and the 2-h time window were chosen to mimic the time the ethanol and sucrose solutions were available in the previous experiments.

On the day of testing, the mice were randomly assigned to the two treatment groups, administered either saline (*n* = 15) or 0.1 mg/kg LSD (*n* = 14) i.p. 3 h after the start of the dark period, and immediately after the injections, given access to pre-weighed portions of food and water, which were then measured again after 2 h. All the mice had received an injection prior to the testing day to habituate them to the procedure.

### Intracranial self-stimulation

#### Surgery

Total of 20 mice (aged 10 weeks at the time of the operation) underwent the implantation surgery. Shortly, a craniotomy was performed under isoflurane (Vetflurane 1000 mg/g, Virbac Animal Health, Carros, France) anaesthesia, with the mice attached to a stereotaxic frame. A 6-mm long (cut below the pedestal), 0.008-inch diameter, 2-channel (bipolar), stainless steel electrode (MS303/2-B/SPC, Plastic One, Roanoke, VA, USA) was implanted into the right side of the head (coordinates −1.6 AP, −1.0 ML, −5.3 DV, mm relative to bregma) targeting the medial forebrain bundle. Two small anchor screws were attached to the skull. The implants were embedded in dental cement, and the wounds were closed with sutures. Carprofen (5 mg/kg; Norocarp Vet 50 mg/ml, Norbrook Laboratories Ltd., Monaghan, Ireland) and buprenorphine (0.05 mg/kg; Temgesic 0.3 mg/ml; Indivior Ltd., Chesterfield, VA, USA) were used for post-operative analgesia.

#### Apparatus and software

The test apparatus consisted of four operant chambers enclosed in wood-composite sound-attenuating cubicles with ventilation fans (Med Associates Inc., Fairfax, VT, USA). The chambers were equipped with a wheel manipulandum, a metal rod floor, a metal tray for aspen bedding material under the floor, a flexible 18-cm long plastic-coated bipolar cable (305-305 C, Plastics One) and a two-channel commutator (SL2C/SB; Plastics One) to connect the cable to constant current stimulators (PHM-152; Med Associates Inc.). The stimulation parameters, data collection and all test session functions were controlled by a computer running the SOF-700RA-5 software package (Med Associates Inc.).

#### Procedure

The protocol was modified from the discrete-trial current-intensity threshold procedure previously described by Stoker and Markou (2011). For daily sessions, the mice were brought into the experimental room to habituate for a minimum of 30 min before the session start. The training and basal sessions between the testing days were performed 5 days a week with 2-day break during the weekends.

Firstly, the mice were trained to turn the manipulandum on a fixed-ratio 1 schedule of reinforcement. Once the mouse had reached the set acquisition criteria (set at 100 reinforcement stimuli in less than 15 min), it proceeded to be trained in the discrete-trial current-threshold phase.

At the start of each trial, the mouse received a non-contingent stimulus, followed by a 7.5-s time window during which the mouse could respond by turning the wheel to receive a contingent stimulus. A response within the window was considered a positive response, and the lack of responses during the window as a negative response. After a negative response, or after a 2-s period where additional responses had no consequences following a positive response, followed an intertrial interval (ITI) with an average duration of 10 s (varying randomly from 7.5 to 12.5 s). Responses during this period, labelled as ITI responses, resulted in a new ITI as a penalty before the initiation of the next trial with a new non-contingent stimulus. During the training on the current-threshold phase, the durations of the ITI and the penalty caused by the ITI responses were gradually increased to reach the 10-s average (ranging from 1 to 10 s; see Supplemental Table S2 for more detailed description of the training phases used).

Each session consisted of four series of blocks with descending and ascending current intensities, always starting with a descent. At each block, the mouse was presented with five trials, and the direction of current intensity changes was based on the responses in these trials: a descent was reversed to ascent after the mouse responded to less than three out of five non-contingent stimuli in two consecutive trial blocks, whereas three or more out of the five responses in two consecutive blocks reversed the ascent to descent. The current step change was 5 μA between the trial blocks. The initial stimulus was set 20–30 μA higher than the expected threshold value, based on the data from the previous session.

The parameters used for the assessment of the performance in the daily sessions were the session threshold (the mean of thresholds from the four series of blocks; a threshold for each descending and ascending series was defined as the midpoint between the current value in μA at which the subject made three or more responses out of the five stimulus presentations and the value at which the subject made less than three responses), the mean latency time (the time between the non-contingent stimulus and the response) and ITI responses per minute. When the session thresholds remained stable, that is, the standard deviation of the last three daily session thresholds was less than 10% of the last threshold, the mouse proceeded to the drug testing. Eleven out of 20 mice that underwent the surgery reached this criterion for the testing phase; see Supplemental Table S1 for detailed exclusion criteria.

The baseline values for the threshold, latency and ITI responses were calculated from the daily means obtained 3 days prior to each treatment and used as the baseline for the drug effect comparisons.

#### Testing

**Figure 4.**
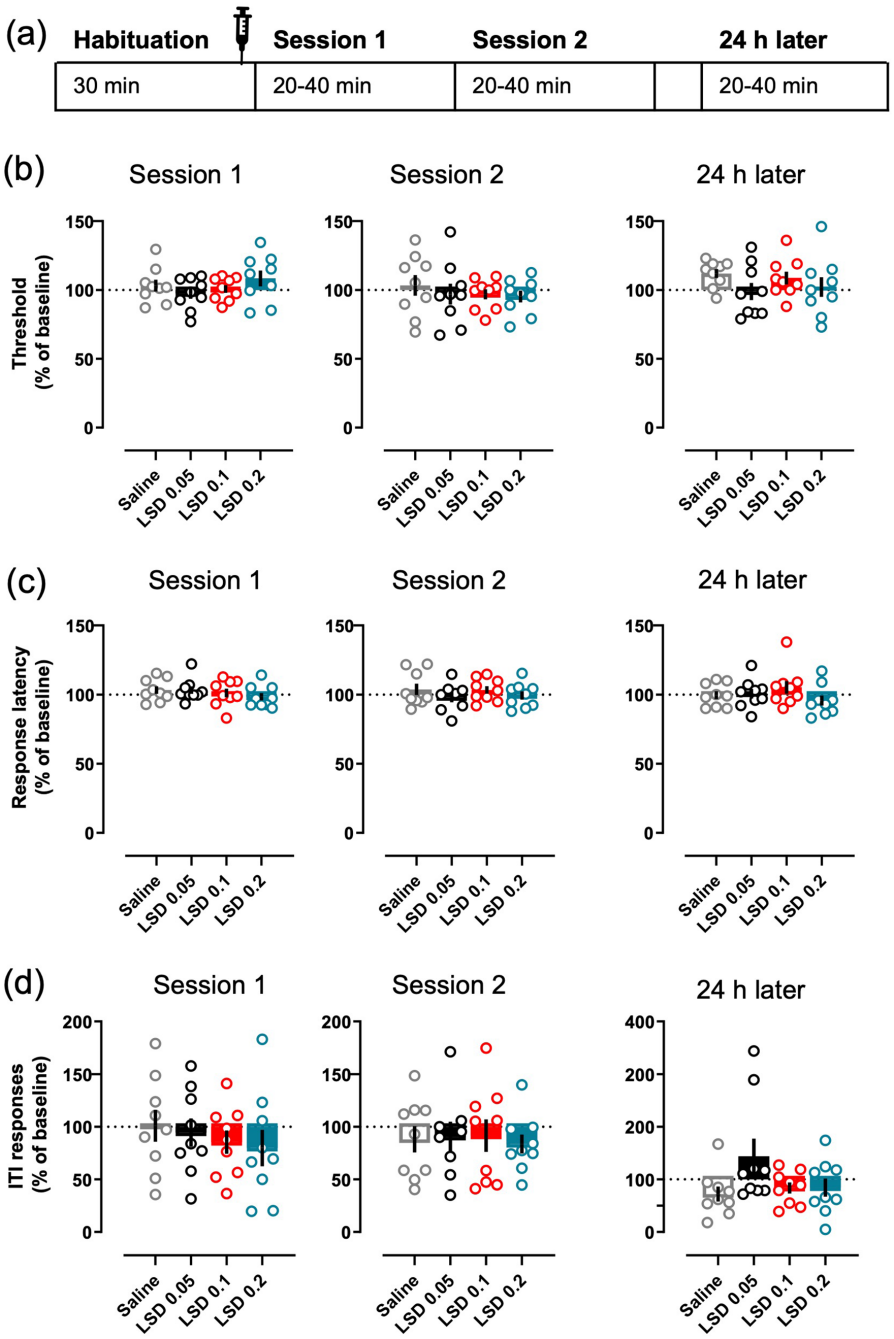
Acute LSD did not affect intracranial self-stimulation. The timing of each treatment session is depicted in the diagram (a), also showing the average times of the sessions. The data for both Sessions 1 and 2, and for the session on the following day, are shown separately for each parameter. None of the tested LSD doses (0.05, 0.1 or 0.2 mg/kg i.p.) affected the main readout, the current threshold (b), neither during the sessions on the day of the administration nor on the following day. The same lack of effects was true with response latency (c) and ITI responding (d). The mean of the 3 days preceding the day of measurement was used as the baseline to which all the parameters were compared to. Data shown as mean ± SEM, circles show individual data points.

**Figure 5.**
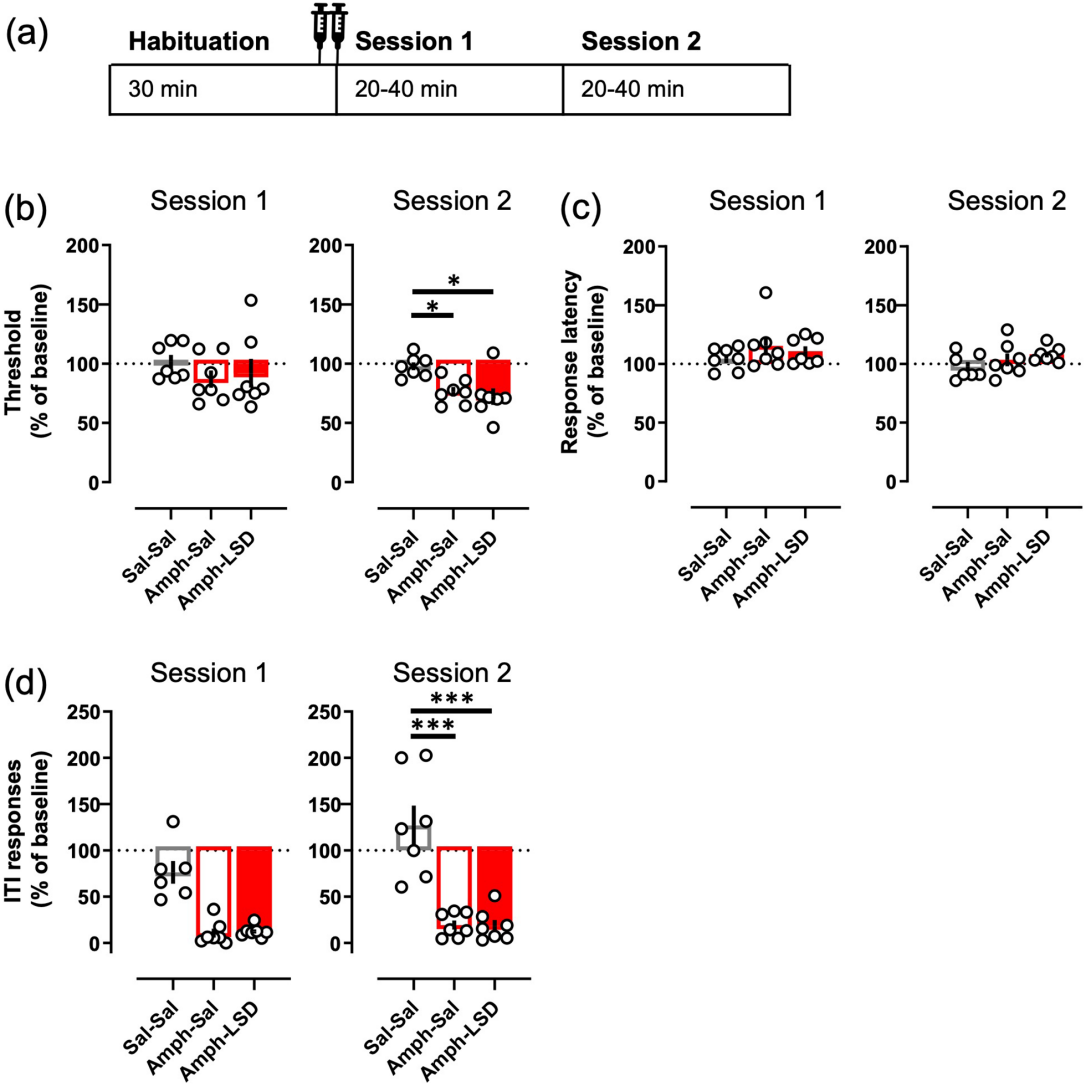
Concurrent administration of LSD did not alter the effects of amphetamine on the ICSS. The two injections (saline + saline; 3.0 mg/kg d-amphetamine + saline; 3.0 mg/kg d-amphetamine + 0.1 mg/kg LSD) were given immediately before the start of the Session 1 as depicted in the diagram (a). Amphetamine significantly decreased the threshold during the Session 2, and simultaneous treatment with LSD did not affect that change to either direction (b). Amphetamine did not have any effect on the response latency alone or with LSD (c) but reduced the ITI responding significantly during the Session 2 (d). The observable decrease during the Session 1 did not reach statistical significance (*p* = 0.067). Data shown as mean ± SEM, circles show individual data points. *p < 0.05, ***p < 0.001.

For the drug testing, each mouse (*n* = 9) received saline and three different doses of LSD (0.05, 0.1 and 0.2 mg/kg i.p.) balanced in time with Latin square design. The mice went through two consecutive sessions immediately after the drug administration (indicated with numbers 1 and 2 in the figures, Figures 4 and 5); this design was used to mitigate the potential effects of the injection and handling stress and to capture longer period of the drugs’ effects. The testing was performed approximately once a week with a minimum of 7 days of washout between the drug treatments. Between the testing sessions, the mice had daily basal sessions on the weekdays and a 2-day break on the weekends; stable baseline threshold (standard deviation of the last three thresholds <10% of the last threshold) was prerequisite for the next treatment.

For the second part of the experiment, the mice (*n* = 7) received one of the following treatment combinations: two injections of saline, saline and 3.0 mg/kg d-amphetamine, or 0.1 mg/kg LSD and 3.0 mg/kg d-amphetamine. Otherwise, the design described above was followed. D-amphetamine worked also as a positive control for the experiment.

After the testing, all the animals were sacrificed by CO_2_ and cervical dislocation, with their brains collected and snap-frozen on dry ice for electrode placement verification (Supplemental Figure S1).

### Data analysis

The statistical analysis was done using InVivoStat software (version 4.2; Clark et al. 2012), and the plots were drawn with Prism 8.1.0 (GraphPad Software, San Diego, CA, USA). In the drinking data, the modification with fixed control value (i.e. the averaged drip control) caused some of the values to become artificial, like negative amounts drank or more than 100% preference. These were all manually corrected to the corresponding limit value (0 or 1) before statistical testing. The proportion responses of the preference data from the drinking experiments were arcsine transformed, whereas the data from ICSS experiment were not, as by the nature of the experiment the proportions were not limited by 100%. All the data were tested for normality and homogeneity of the variance using the normal probability plots and the predicted versus residuals plots, respectively (Bate and Clark 2014: 152–158). Whenever the assumptions were violated, the data were square root transformed, and in the case of persisting violation, a non-parametric variant of the intended test was used. The level of significance was set at 0.05. All the data are shown as means ± SEM, unless otherwise indicated.

For the ethanol and sucrose drinking experiments, the analyses between the treatment groups on the day of drug administration were done with one-way analysis of variance followed by Dunnett’s multiple comparison procedure comparing the two LSD doses to the saline control, or the Kruskal–Wallis test when the parametric assumptions were violated. To assess the differences within the treatment groups and between different measurement points, a two-way repeated measures mixed model approach was used based on the data of the drug treatment day, four prior days as the baseline days and four following days as the follow-up. A full pairwise comparison between all the days was used, and the *p*-values of the planned comparisons between the day before the treatment, the drug day and the following day were adjusted using Holm’s multiple comparison procedure.

The treatment effects on the food and water consumption experiment were analysed using the Student’s *t*-test for the between-group comparisons for the treatment day. Two-way repeated measures mixed model approach was used to analyse the data between the baseline 2 and the drug day, followed by full pairwise comparison and Holm’s adjustment procedure for the planned comparisons between the within-group means on these 2 days.

A full crossover design was used for the ICSS experiment; therefore, each parameter was analysed using one-way analysis of variance with the different treatment combinations or doses as the treatment factor, and animals and days as blocking factors to account for the within-animal variability (Bate and Clark 2014: 59–60, 251–252). In the case of statistically significant main effect, the omnibus test was followed with a Tukey’s HSD (honestly significant difference) multiple comparison procedure. As the animals went through two consecutive sessions after each drug treatment, for each measured parameter, the session outcome was analysed against the corresponding session of the other treatments. The effects on threshold, latency and ITI responses were analysed as proportional changes compared to the mean of the three precedent days.

## Results

### Intermittent ethanol drinking

The mice reached mean ethanol preference of more than 50% by the week 4 (Figure 1(b); 53% ± 11% (mean ± 95% CI) preference on the fourth day). On the treatment day, both LSD groups drank less ethanol than the saline control group (g/kg; Figure 1(c); main effect *F*(2, 24) = 3.53, *p* = 0.0045) with the LSD 0.1 mg/kg group drinking significantly less than the control group (−83%, Dunnett’s test *p* = 0.026). Similarly, when comparing the intake levels within each group, the LSD 0.1 mg/kg group drank significantly less on the treatment day than on the previous day (*p* = 0.0045), whereas the changes in the other two groups were not significantly different (*p* > 0.1). We observed neither significant differences between the last baseline day and the first follow-up day nor between the treatment day and the follow-up day in any of the groups (*p* > 0.1). There were no differences between the groups in total fluid intake (Figure 1(f); *F*(2, 24) = 0.57, *p* = 0.57) on the day of the treatment, and a pairwise analysis after the significant day main effect (*F*(8, 192) = 6.6, *p* < 0.0001) in repeated measures analysis showed that the decrease from the baseline to the drug day was not significant in the LSD groups (Holm’s *p* > 0.1). There was a significant difference between the saline group and the 0.1 mg/kg LSD group in the ethanol preference on the day of the treatment (*F*(2, 24) = 10.03, *p* = 0.0007, Dunnett’s test, *p* = 0.003), and a trend between the saline and the lower-dose LSD group (Dunnett’s test, *p* = 0.05).

A repeated measures analysis of the preference data revealed a significant Treatment × Day interaction (*F*(16, 192) = 1.87, *p* = 0.025). Further analysis showed that only the 0.1 mg/kg dose of LSD significantly decreased the ethanol preference on the treatment day (Figure 1(g); −40% compared to the previous day, Holm’s *p* = 0.009), whereas the preference stayed the same with the 0.05 mg/kg dose of LSD (+1%), and even increased in the control group although in a statistically non-significant manner (+25%, Holm’s *p* = 0.28). This was reflected to some extent in the water consumption, where the slight increase in LSD 0.1 mg/kg group and the decrease in the control group (Figure 1(e)) resulted in a statistically significant difference *F*(2, 24) = 6.4, *p* = 0.0059, Dunnett’s test *p* = 0.0029) on the treatment day, but no statistically significant differences were observed when comparing the changes within the groups between the baseline, treatment and follow-up days (Treatment *F*(2, 24) = 0.56, *p* = 0.58; Day *F*(8, 192) = 1.71, *p* = 0.09).

### Intermittent sucrose drinking

Contrasting with the ethanol experiment, the mice preferred the sucrose solution from the first day onwards (Figure 2(b); 97% ± 4% (mean ± 95% CI) preference on the fourth day). The treatment with LSD slightly decreased sucrose intake in both groups (LSD 0.05 −13%; LSD 0.1 −14%) as compared to saline treatment, but the differences between the three groups were neither statistically significant (g/kg; Figure 2(c); *F*(2, 26) = 1.72, *p* = 0.2), nor decreased from their own baselines (*p* > 0.1). On the contrary, a significant increase in water intake was observed in all groups on the day of the treatment (0.6 ± 0.09 ml in all groups; Figure 2(e); Day main effect *F*(8, 208) = 71.3, *p* < 0.0001, Holm’s *p* < 0.0001 in each group between the baseline and treatment days). While this increase did not alter the total fluid intake (Figure 2(d)), it was large enough to cause significant drop in sucrose preference in every group (Figure 2(g); Day main effect *F*(8, 208) = 9.5, *p* < 0.0001, Holm’s corrected *p* ⩽ 0.005 in every group between the baseline and treatment days).

Because this increase in water intake caused uncertainty on the other results – the increase potentially masked some other effects, like potential changes in sucrose preference caused by LSD – the initial test was followed up using an otherwise identical design but with additional repeated saline injections (Figure 3(a)). The daily saline injections lowered the water intake on Days 26–28 to the level of the original drinking phase as shown on Day 20 (Figure 3(c)). As in the previous test (Figure 2(c)), no significant differences between the treatment groups were observed in sucrose consumption (g/kg; Figure 3(f); *F*(2, 26) = 0.61, *p* = 0.55). No differences between the treatment groups were observed in water consumption (ml; Figure 3(c); *H*(2) = 1.19, *p* = 0.55) either. These were echoed by the lack of changes in sucrose preference (Figure 3(e); *F*(2, 26) = 1.2, *p* = 0.31) and in total fluid intake (Figure 3(d); *F*(2, 26) = 0.35, *p* = 0.71). Repeated measures analysis did neither reveal any significant treatment effects in sucrose consumption (Treatment *F*(2, 26) = 1.16, *p* = 0.33), water intake (Treatment *F*(2, 26) = 0.02, *p* = 0.97) nor in the total fluid intake (Treatment *F*(2, 26) = 1.12, *p* = 0.34).

### Eating and drinking behaviours

**Figure 6.**
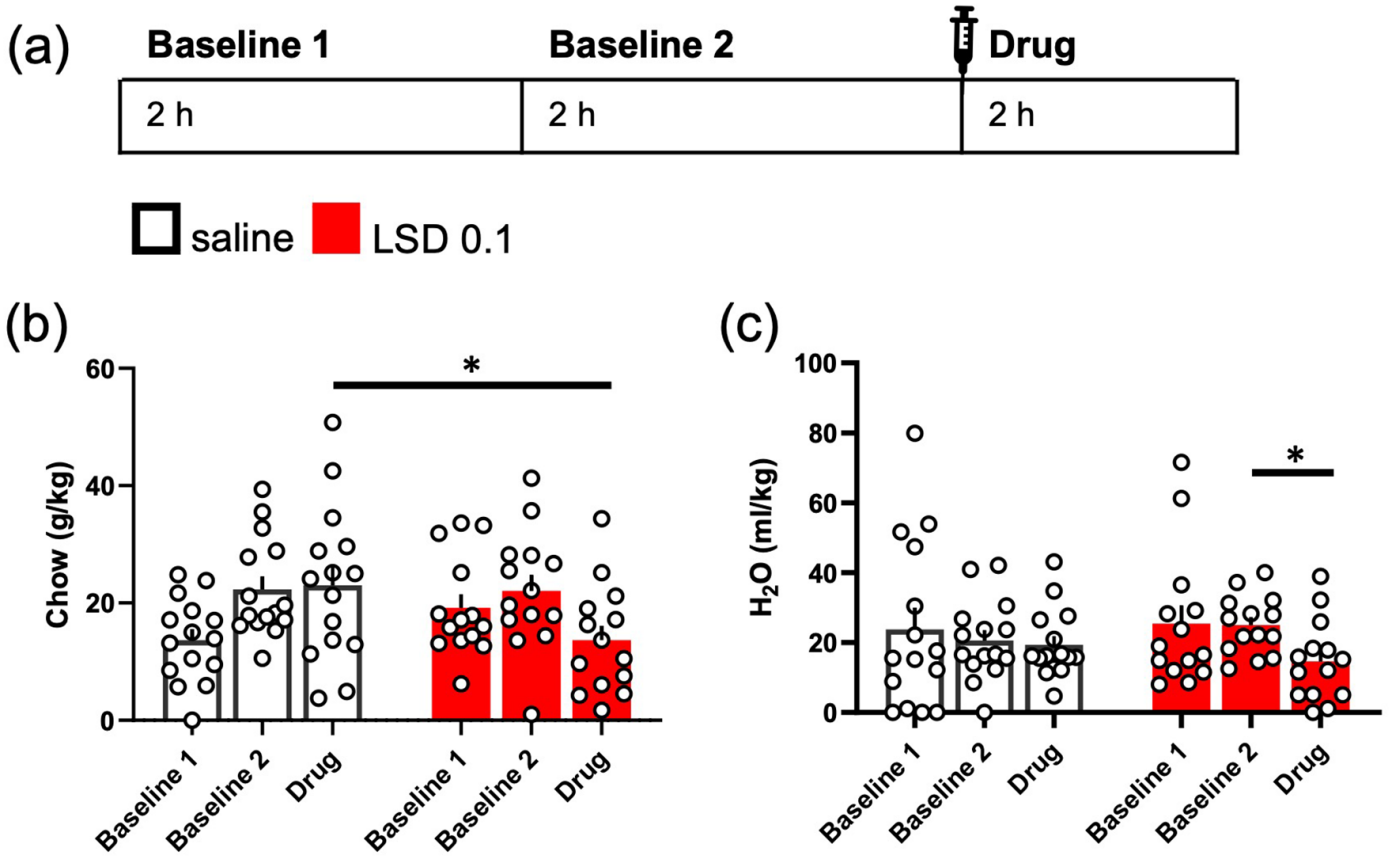
LSD acutely reduced normal eating and drinking behaviour. The design of the experiment is depicted in the diagram (a). Eating and drinking of single-housed mice were measured for 2 h on two consecutive days, mimicking the timing of the intermittent drinking experiments. LSD at the dose of 0.1 mg/kg acutely reduced the amount of chow (g/kg) the mice ate when comparing to the saline control group, but not when compared to the group’s own baseline (b). The 2-h water intake after LSD treatment was lower than the group’s own baseline (Baseline 2) but did not significantly differ from that of the saline control group (c). Data shown as mean ± SEM, circles in (b) and (c) show individual data points. *p < 0.05.

No difference in the baseline eating was observed between the two treatment groups (g/kg, Baseline 2; Figure 6(b); *t*(27) = −0.009, *p* = 0.99). The acute treatment with 0.1 mg/kg LSD decreased chow consumption of the mice when compared to the saline control (Figure 6(b); *t*(26, 92) = 2.081, *p* = 0.0471). When compared to the groups’ own baselines (Baseline 2), no within-group changes were observed with either treatment (Day main effect *F*(1, 27) = 2.98, *p* = 0.0959).

Similarly, there was no significant difference in water consumption baseline levels (Baseline 2; Figure 6(c); *t*(27) = −1.346, *p* = 0.1895). LSD treatment did not affect the drinking behaviour acutely in comparison to the saline control (*t*(27) = 1.035, *p* = 0.3098). However, the decrease within the LSD treatment group, when compared to the baseline, was statistically significant (Day *F*(1, 27) = 5.42, *p* = 0.0276, Baseline 2 vs Drug *p* = 0.018), whereas there was no change within the saline control group (Baseline 2 vs Drug *p* = 0.6578).

### Intracranial self-stimulation

None of the tested LSD doses produced any changes on the current-intensity threshold during the first (Figure 4(b); *F*(3, 21) = 1.2, *p* = 0.33) nor the second session (*F*(3, 21) = 0.73, *p* = 0.55); the mean thresholds remained at the baseline level after the LSD treatments (Session 1: 97.4 – 108.36%; Session 2: 96.97 – 95.29%) as was the case with the saline control (Session 1: 103.2%; Session 2: 103.21%). No differences between the treatments were observed in the response latency in either session (Figure 4(c); Session 1: *F*(3, 21) = 0.72, *p* = 0.55; Session 2: *F*(3, 21) = 1.82, *p* = 0.17), nor in the ITI responses (Figure 4(d); Session 1: *F*(3, 21) = 0.61, *p* = 0.31; Session 2: *F*(3, 21) = 0.47, *p* = 0.71). Analysis of the corresponding data of the next session, 24 h after the treatment, did not reveal any significant effects in any of the tested parameters (Figure 4(b)–(d); Threshold *F*(3, 21) = 0.75, *p* = 0.54; Latency *F*(3, 21) = 1.47, *p* = 0.25; ITI responses *F*(3, 21) = 2.37, *p* = 0.1).

The amphetamine treatment, with or without the concurrent LSD, did not affect the current threshold during the first session (Figure 5(b); *F*(2, 10) = 1.00, *p* = 0.40), but lowered the threshold significantly on the second session (*F*(2, 10) = 7.76, *p* = 0.0093; Sal–Sal vs Amph–Sal *p* = 0.03). The simultaneous treatment with 0.1 mg/kg LSD had no observable effect on this decrease to either direction (Sal–Sal vs Amph–LSD *p* = 0.011; Amph–Sal vs Amph–LSD *p* = 0.85). The amphetamine treatment, again with or without LSD, had no effect on the response latency on either session (Figure 5(c); Session 1: *F*(2, 10) = 0.92, *p* = 0.37; Session 2: *F*(2, 10) = 1.3, *p* = 0.32). However, the amphetamine treatment decreased the ITI responding highly significantly compared to the saline control during the second session (Figure 5(d); *F*(2, 10) = 31.13, *p* < 0.0001; Sal–Sal vs Amph–Sal *p* < 0.0001). Here again, the simultaneous LSD treatment did not affect the decrease in any observable way (Sal–Sal vs Amph–LSD *p* < 0.0001; Amph–Sal vs Amph–LSD *p* = 0.98). After both amphetamine-containing treatments, the ITI responding was visibly lower than after the saline treatment during the first session (Figure 5(d); 14% and 16% from the saline control, respectively), but the statistical analysis only revealed a trend (*F*(2, 10) = 3.6, *p* = 0.067).

## Discussion

The present set of experiments sought to study the effects that acute administration of LSD might have on ethanol consumption of mice in a binge-like intermittent access schedule, and further to investigate the potential reasons and generalizability of the observed effects to other positive reinforcers. To our knowledge, it is also the first study to investigate the effects of classic psychedelic drugs on binge-like ethanol drinking. We found that, in this experimental setting, acute 0.1 mg/kg LSD treatment reduced the 2-h ethanol intake. No further significant effect was observed on the following days of ethanol availability. This contrasts with the prolonged effects shown by Alper et al. (2018), who reported a single 0.05 mg/kg dose of LSD – a dose without significant effects in the present study – to significantly reduce ethanol consumption in mice for more than 40 days. It could be argued that in our study, the level of ethanol intake of the 0.1 mg/kg LSD treatment group stays at a lower level after the treatment (Figure 1(d)) in a similar fashion, but since the divergence was not statistically significant, this effect was not pursued further. The differences in the observed effects between the studies could stem from the different ethanol access schedules, leading to differing drinking behaviours: the study of Alper et al. (2018) had ethanol available for 24 h for five consecutive days, whereas our study had the access to ethanol limited to 2-h periods on four consecutive days, reminding more quick, binge-like consumption versus the more episodic, but stable consumption in the 24-h model (Rhodes et al., 2005; Thiele and Navarro, 2014). Furthermore, Meinhardt et al. (2020) investigated the effects of psilocybin and LSD using a rat model of alcohol relapse drinking and also failed to see any significant long-lasting changes in ethanol intake; only a repeated dosing with psilocybin when administered day before and after the start of ethanol availability significantly reduced relapse drinking. Thus, it is possible that LSD and psychedelics, in general, have different effects depending on the ethanol drinking behaviour. When comparing their effects, it is also good to note that LSD and psilocybin have differing pharmacological profiles: psychedelics are commonly thought to act through 5-HT_2A_ receptor agonism, but they all also have effects on the other serotonin receptors and beyond. LSD is set apart from the other by its significant agonistic affinity to dopamine receptors, but it also has greater affinity to adrenergic receptors compared to psilocin, the active, dephosphorylated form of psilocybin (Halberstadt and Geyer, 2011). The roles of these other receptors in the effects of psychedelic drugs, especially in their potential therapeutic applications, are still widely unknown.

More similar, while not identical, to our experimental design are two recent studies investigating the effects of the hallucinogenic 5-HT_2A/2C_ receptor agonist, DOI: Oppong-Damoah et al. (2019) reported that 3.0 mg/kg DOI decreases voluntary ethanol intake in mice in an intermittent drinking schedule both at 1- and 24-h measurement points, and Berquist and Fantegrossi (2021), using the same schedule in rats, showed that 1.0 mg/kg DOI reduces drinking at 1-, 2- and 24-h timepoints. While no further follow-up observations were reported, and the prolonged effects beyond 24 h cannot be therefore compared, the acute effects resemble our findings: the latter study reported 1.0 mg/kg DOI to have caused decreases of identical level (approximately 80% reduction compared to the saline control) in their 1-h measurement as our 0.1 mg/kg LSD caused in the 2-h measurement window (Berquist and Fantegrossi, 2021). Similar results were earlier reported by Maurel et al. (1999, 2000), who reproducibly showed that DOI decreases 12-h ethanol intake in a two-bottle choice voluntary home cage drinking setting in AA rats.

A potential mechanism for the reduced ethanol consumption could be that LSD somehow blunts the rewarding effects of ethanol intake. To further investigate this possibility, we replicated the intermittent binge-like drinking experiment but replaced the ethanol with a natural reinforcer using 10% sucrose solution. Before considering the main findings, the unexpected increase in water intake observed in all three groups after the injections (Figure 2(e) and (g)) warrants a note. Based on our earlier experiences, no need for injection habituation before the treatment day was considered necessary, and the increased water intake was therefore unexpected. As the effect was seen in all three groups, and again with the repeated saline injections in the repetition phase of the experiment, we considered the effect to stem either from the saline or the injection itself. Injection of hypertonic saline is known to increase water intake (McKinley et al., 2008), but our use of different batches of saline in the different parts of the experiment should negate the possibility of non-physiological saline since the effect persisted. Acute stressors have been previously shown to increase water intake in a similar two-bottle choice setup (Cozzoli et al., 2014), and since our effect levelled out after repeated, daily injections, an injection stress is a most likely explanation for the observed effect here. The social isolation in single cages needed for the experimental designs of the present study might be part of the effect as it is known to exacerbate stress-related behavioural and physiological effects (Valzelli, 1973). However, social isolation also gives male mice an opportunity to fulfil some sex-specific territorial needs (Kappel et al., 2017) and is also known to increase pain threshold (Puglisi-Allegra and Oliverio, 1983), and, therefore, cannot be singled out as the main cause for the observed stress-like effect on drinking. Habituation for injections was performed before repeating the sucrose test to minimize the potential of the suspected injection stress masking LSD-induced effects on sucrose preference. In the end, with evened-out water intake, neither tested dose of LSD affected the sucrose intake nor the preference, questioning the possibility of the proposed reward-attenuating effects of LSD as the effect was not generalized to another reinforcing solution. We are unaware of earlier investigations of acute effects of psychedelics or 5-HT_2A_ agonists on similar binge-like sucrose drinking, but Maurel et al. (2000) showed that 3.0 mg/kg DOI decreased ethanol intake but not the intake of simultaneously present water or sweet sucrose- or saccharin-containing solutions, which resembles our findings.

The theory of reward-attenuation by LSD was further challenged by the outcomes of our ICSS experiment, where none of the tested LSD doses altered the current-intensity threshold, the main reward-linked measure of the procedure (Markou and Koob, 1992). On the other hand, a recent article using rats and frequency-rate procedure of ICSS showed some stimulation-depressing effects by LSD, psilocybin and mescaline treatments, especially when LSD was used at a similar dose range as in our study (Sakloth et al., 2019). This in turn could support the suggested reward-attenuation mechanism. However, due to differences in methodology, our findings are not directly comparable. Observations by Sakloth et al. (2019) are in line with earlier results showing the selective 5-HT_2A_ agonist TCB-2 to increase the current-intensity threshold, or to be aversive (Katsidoni et al., 2011). Still, in line with LSD not affecting the threshold-lowering effects of amphetamine in our study, neither Katsidoni et al. (2011) nor Sakloth et al. (2019) observed any of the tested psychedelics or 5-HT_2A_ agonists to modify the rewarding effects of psychostimulants, cocaine and methamphetamine, respectively.

Another possible mechanism is the opposite direction of reward modulation, that is, enhancement of aversion instead of dampening reward: while we did not observe LSD itself to be aversive, it could possibly potentiate aversive subjective experiences caused by ethanol (Stewart and Grupp, 1986), something that might not be present with sucrose or psychostimulants. This theory was not tested in the present study, but the findings of Sakloth et al. (2019) would rather point towards the opposite direction as they reported LSD to attenuate the negative effects of the kappa opioid receptor agonist U69,593. However, this attenuation was achieved only after a 7-day repeated LSD treatment. Since there are reports of repeated LSD administration producing conditioned place preference, that is, being rewarding in rodents (Meehan and Schechter, 1998; Parker, 1996), and – as mentioned earlier – since the largest reducing effect on ethanol drinking is seen only with repeated psilocybin doses in Meinhardt et al. (2020), the differences between administration schedules of psychedelics and their effects on reward-linked behaviours might be meaningful and worth looking into in the future.

In theory, the reduced ethanol intake we observed could be due to more general behavioural disruption caused by the LSD treatment. The observed uninterrupted sucrose drinking, together with the non-significant changes in total fluid intake in the ethanol experiment, implicate that the reduced ethanol intake is not caused by impairment of the animals’ ability to drink. In addition, as already indicated by earlier operant studies with psychedelics, rodents can perform complex behaviours under the acute influence of LSD (Elsilä et al., 2020; Hirschhorn and Winter, 1971). In the present study, this is supported by the lack of alterations in the performance-indicator readouts like response latency and ITI responses (Markou and Koob, 1992) in the ICSS. LSD rendering the mice somehow incapacitated is, therefore, not an explanation for the decreases in ethanol intake, which is further supported by the non-affected locomotor activity results by Alper et al. (2018).

Since ethanol is a caloric liquid, any changes to homeostatic consummatory behaviour might cause changes in ethanol intake as well. As the food intake was not measured in either intermittent drinking setting, a separate experiment was executed, and the results show small but significant decreases in both eating and water consumption after the administration of 0.1 mg/kg LSD. Previous findings about the effects of psychedelics and related 5-HT_2A_ agonists on food intake are mixed: Maurel et al. (1999) measured food intake during the voluntary ethanol consumption in home cages and did not observe significant changes after the administration of DOI, whereas Berquist and Fantegrossi (2021) showed DOI to decrease food consumption in a similar ethanol-drinking setting, with significant effects observable at 1-, 2- and 24-h measurement points. An experimental design closer to ours, measuring 2-h food intake after acute LSD treatment, showed similar decreases in a dose-dependent manner (Hamilton and Wilpizeski, 1961). While our findings support the idea of acute diminution of consummatory behaviours, it is noteworthy that LSD treatment did not completely eliminate either eating or drinking within the measurement period. This together with the lack of effects in sucrose intake cause doubt in ubiquitous and strong effects on consummatory behaviours.

The main caveat of the present study is the use of only male mice. Mice are known to have sex differences in their ethanol intake, and in intermittent access designs as was used in the present study, female mice tend to drink more than male mice (Rhodes et al., 2005; Tylš et al., 2016). In addition, psychedelic compounds are known to have differing effects in male and female rodents. Female rats are less sensitive to locomotor and thigmotaxis-related effects of LSD (Páleníček et al., 2010), and sex appears to contribute on the disruption of pre-pulse inhibition by psychedelics both in rats and mice (Páleníček et al., 2010; Vohra et al., 2021). Furthermore, while an example of a different drug class, the glutamate N-methyl-D-aspartate receptor inhibitor ketamine might affect ethanol binge drinking differently in male and female mice (Crowley et al., 2019). Clearly there is a need for greater consideration of sex as a biological variable in the future psychedelics research using rodent models.

While the aim of the present study was not to mimic the therapeutic setting used in the current clinical investigations, a short comparison is justified. Different forms of therapy and guidance are essential elements in the current clinical trials investigating therapeutic potential of psychedelic compounds. They are used to build trust and rapport between the trial staff and patients, to enhance the motivation of the patients towards therapeutic outcomes and to help them utilize their experiences during and after the therapy session (dos Santos and Hallak, 2020; Johnson et al., 2008). Therapies like these are very difficult, maybe even impossible, to model in rodents, and the complete absence of such elements in our approach might be one of the reasons why no long-lasting effects were observed. The lack of observable effects in the study by Meinhardt et al. (2020), more specifically in the experiments where the administration of psychedelics was designed to resemble the schedules used in clinical trials, implies that the timing of the drug administration alone might not be the cause for the differences. Many participants, for example, in the Johns Hopkins’ tobacco cessation trial reported that they had learned something about themselves, their reasons to smoke, or their relation to their environment, and that this novel self-discovery was elemental to their abstinence (Noorani et al., 2018). Therefore, building a rodent experiment to include a possibility for the animals to learn something new about the self-administrable ethanol, for example, through some form of reward discounting (neglecting availability and increasing aversivity), might give the research more translational validity.

Another potentially interesting translational line of inquiry, stemming from the human imaging findings, is related to the default mode network, a functional brain network classically related not only to internally oriented thoughts (Buckner et al., 2008), but also potentially important for social interactions (Yeshurun et al., 2021). In human imaging studies, psychedelics have been repeatedly shown to decrease the integrity of this network, and this has been postulated to correlate with the known changes in self- and social-processing and potentially to be a key element in their therapeutic efficacy (Vollenweider and Preller, 2020). Dysfunctions of the default mode network have also been implicated in addiction, especially in relation to drug craving and relapse (Zhang and Volkow, 2019), although functional connectivity between the nodes of the default mode network have also been shown to be altered by acute alcohol intake (Fang et al., 2021). Crucially from the rodent-to-human translational perspective, psilocybin has been shown to alter the functional connectivity between hub areas of the mouse default mode network homologue (Grandjean et al., 2021).

One of the main advantages in using rodent models in psychedelic research is the possibility of studying molecular mechanisms with tools not available in humans. For example, metabotropic glutamate (mGlu) receptors offer an interesting target to further clarify the mechanisms of action of psychedelics. Changes in mGlu receptor system have been implicated in intermittent ethanol intake in mice, including increased mGlu_2/3_ receptor-mediated long-term depression in the subset of pyramidal cells of the medial prefrontal cortex (Joffe et al., 2021), increased mGlu_5_ receptor expression in the nucleus accumbens (Cozzoli et al., 2012), and, furthermore, mGlu2/3 agonist reduces alcohol seeking in mice (Windisch and Czachowski, 2018). Beyond the previously suggested interplay between the mGlu_2/3_ receptors and 5-HT_2A_ receptor (González-Maeso et al., 2008; Toneatti et al., 2020), it was recently shown that psilocybin can recover the alcohol dependence-induced downregulation of mGlu_2_ expression in the infralimbic cortex in rats, and that this recovery was correlated with significant reductions in craving-like alcohol-seeking behaviours (Meinhardt et al., 2021). On the basis of these recent discoveries, investigating the potential of psychedelics to modulate the mGlu receptor effects would be warranted and further extended to understand whether pharmacological modulation of both mGlu and serotonergic receptor systems together would enhance the treatment efficacy in alcohol addiction.

## Conclusion

Taken together, the present study is the first to show the acute reducing effects of LSD administration on binge-like ethanol drinking in mice. As acute LSD did not affect binge-like sucrose drinking or brain self-stimulation reward, we assume that the effects on the ethanol intake are not caused by any reward-attenuating effects of LSD. Discrepancies between our and some of the earlier findings warrant further investigation, focusing on the effects of the drug administration timing and schedules of ethanol availability. We also conclude that based on our results, the acute decrease in ethanol consumption might be, in part, caused by modulation of general consummatory behaviours, but it can hardly alone explain the changes and needs still more systematic research.

## Supplemental Material

sj-pptx-1-jop-10.1177_02698811221104641 – Supplemental material for Effects of acute lysergic acid diethylamide on intermittent ethanol and sucrose drinking and intracranial self-stimulation in C57BL/6 mice Click here for additional data file. Supplemental material, sj-pptx-1-jop-10.1177_02698811221104641 for Effects of acute lysergic acid diethylamide on intermittent ethanol and sucrose drinking and intracranial self-stimulation in C57BL/6 mice by Lauri V Elsilä, Juliana Harkki, Emma Enberg, Alvar Martti, Anni-Maija Linden and Esa R Korpi in Journal of Psychopharmacology
