# Supplementary material for: Effects of acute lysergic acid diethylamide on intermittent ethanol and sucrose drinking and intracranial self-stimulation in C57BL/6 mice
Source: J Psychopharmacol. 2022 Jun 13;36(7):860–74. doi: 10.1177/02698811221104641 (PMC9247434; doi:10.1177/02698811221104641)
Supplement: sj-pptx-1-jop-10.1177_02698811221104641 – Supplemental material for Effects of acute lysergic acid diethylamide on intermittent ethanol and sucrose drinking and intracranial self-stimulation in C57BL/6 mice [file sj-pptx-1-jop-10.1177_02698811221104641.pptx]

## Slide 1
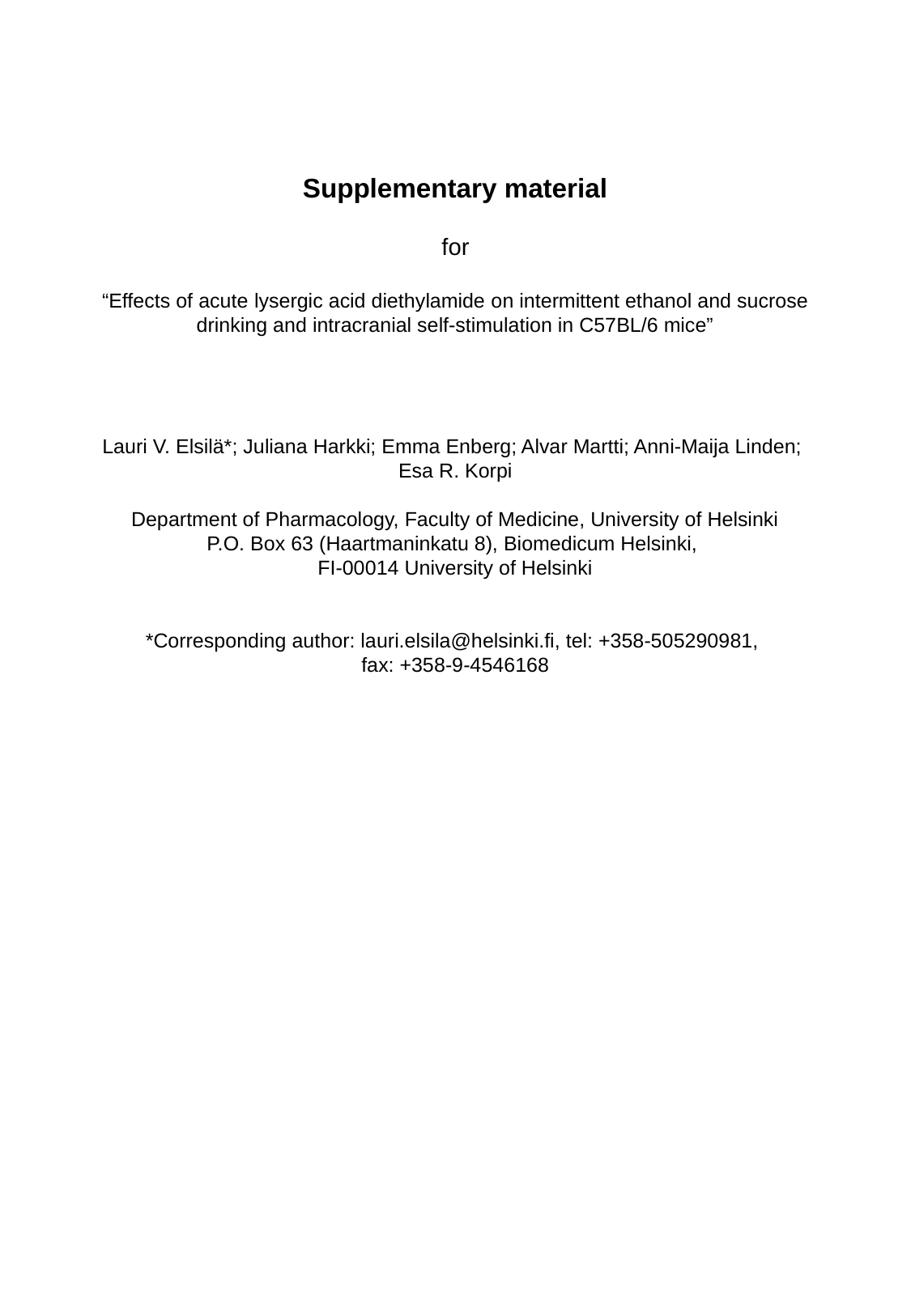

Supplementary material
for
“Effects of acute lysergic acid diethylamide on intermittent ethanol and sucrose drinking and intracranial self-stimulation in C57BL/6 mice”
Lauri V. Elsilä*; Juliana Harkki; Emma Enberg; Alvar Martti; Anni-Maija Linden;
Esa R. Korpi
Department of Pharmacology, Faculty of Medicine, University of Helsinki
P.O. Box 63 (Haartmaninkatu 8), Biomedicum Helsinki,
FI-00014 University of Helsinki
*Corresponding author: lauri.elsila@helsinki.fi, tel: +358-505290981,
fax: +358-9-4546168

## Slide 2
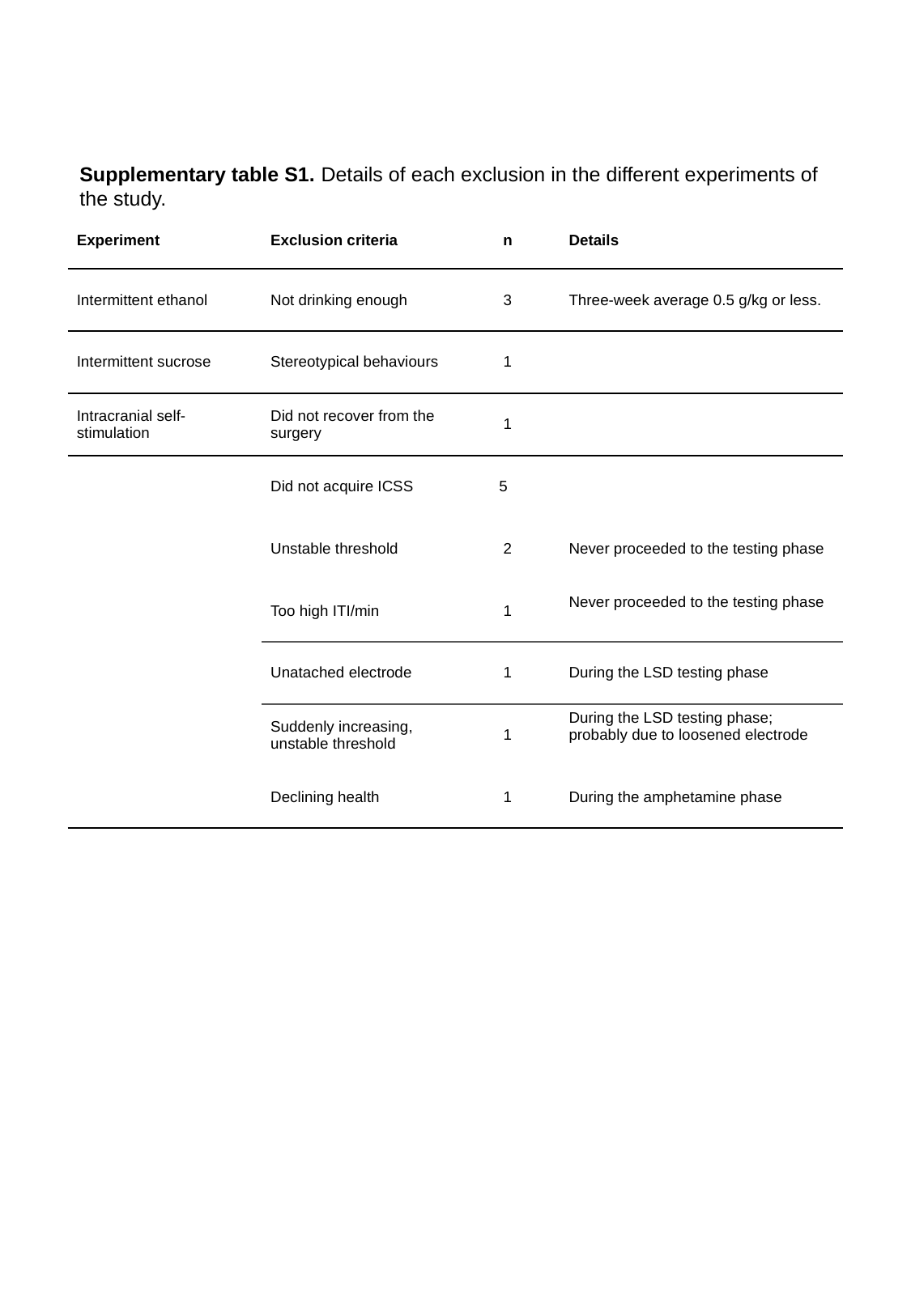

Supplementary table S1. Details of each exclusion in the different experiments of the study.
| Experiment | Exclusion criteria | n | Details |
| --- | --- | --- | --- |
| Intermittent ethanol | Not drinking enough | 3 | Three-week average 0.5 g/kg or less. |
| Intermittent sucrose | Stereotypical behaviours | 1 | |
| Intracranial self-stimulation | Did not recover from the surgery | 1 | |
| | Did not acquire ICSS | 5 | |
| | Unstable threshold | 2 | Never proceeded to the testing phase |
| | Too high ITI/min | 1 | Never proceeded to the testing phase |
| | Unatached electrode | 1 | During the LSD testing phase |
| | Suddenly increasing, unstable threshold | 1 | During the LSD testing phase; probably due to loosened electrode |
| | Declining health | 1 | During the amphetamine phase |

## Slide 3
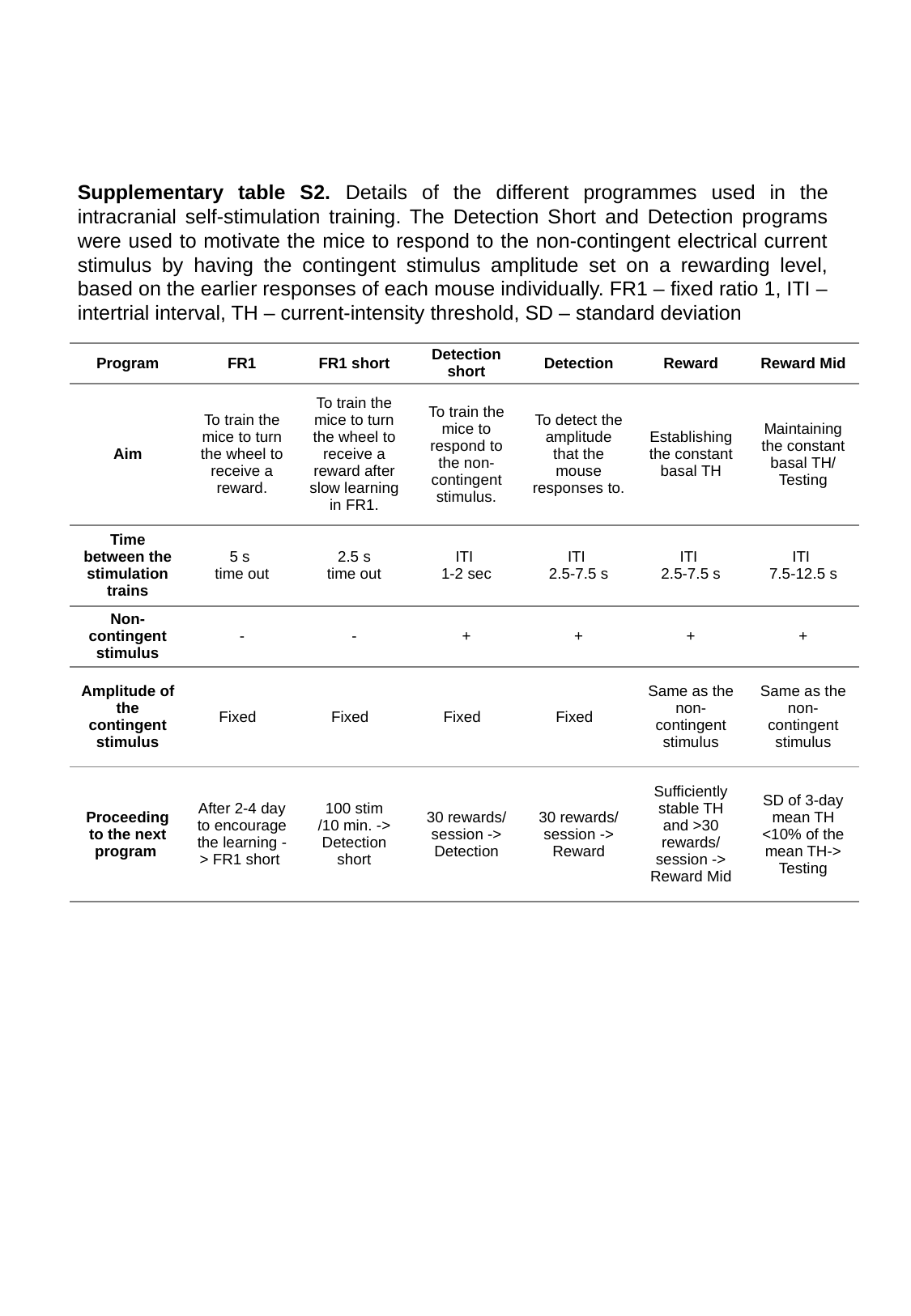

Supplementary table S2. Details of the different programmes used in the intracranial self-stimulation training. The Detection Short and Detection programs were used to motivate the mice to respond to the non-contingent electrical current stimulus by having the contingent stimulus amplitude set on a rewarding level, based on the earlier responses of each mouse individually. FR1 – fixed ratio 1, ITI – intertrial interval, TH – current-intensity threshold, SD – standard deviation
| Program | FR1 | FR1 short | Detection short | Detection | Reward | Reward Mid |
| --- | --- | --- | --- | --- | --- | --- |
| Aim | To train the mice to turn the wheel to receive a reward. | To train the mice to turn the wheel to receive a reward after slow learning in FR1. | To train the mice to respond to the non-contingent stimulus. | To detect the amplitude that the mouse responses to. | Establishing the constant basal TH | Maintaining the constant basal TH/ Testing |
| Time between the stimulation trains | 5 s time out | 2.5 s time out | ITI 1-2 sec | ITI 2.5-7.5 s | ITI 2.5-7.5 s | ITI 7.5-12.5 s |
| Non-contingent stimulus | - | - | + | + | + | + |
| Amplitude of the contingent stimulus | Fixed | Fixed | Fixed | Fixed | Same as the non-contingent stimulus | Same as the non-contingent stimulus |
| Proceeding to the next program | After 2-4 day to encourage the learning -> FR1 short | 100 stim /10 min. -> Detection short | 30 rewards/ session -> Detection | 30 rewards/ session -> Reward | Sufficiently stable TH and >30 rewards/ session -> Reward Mid | SD of 3-day mean TH <10% of the mean TH-> Testing |

## Slide 4
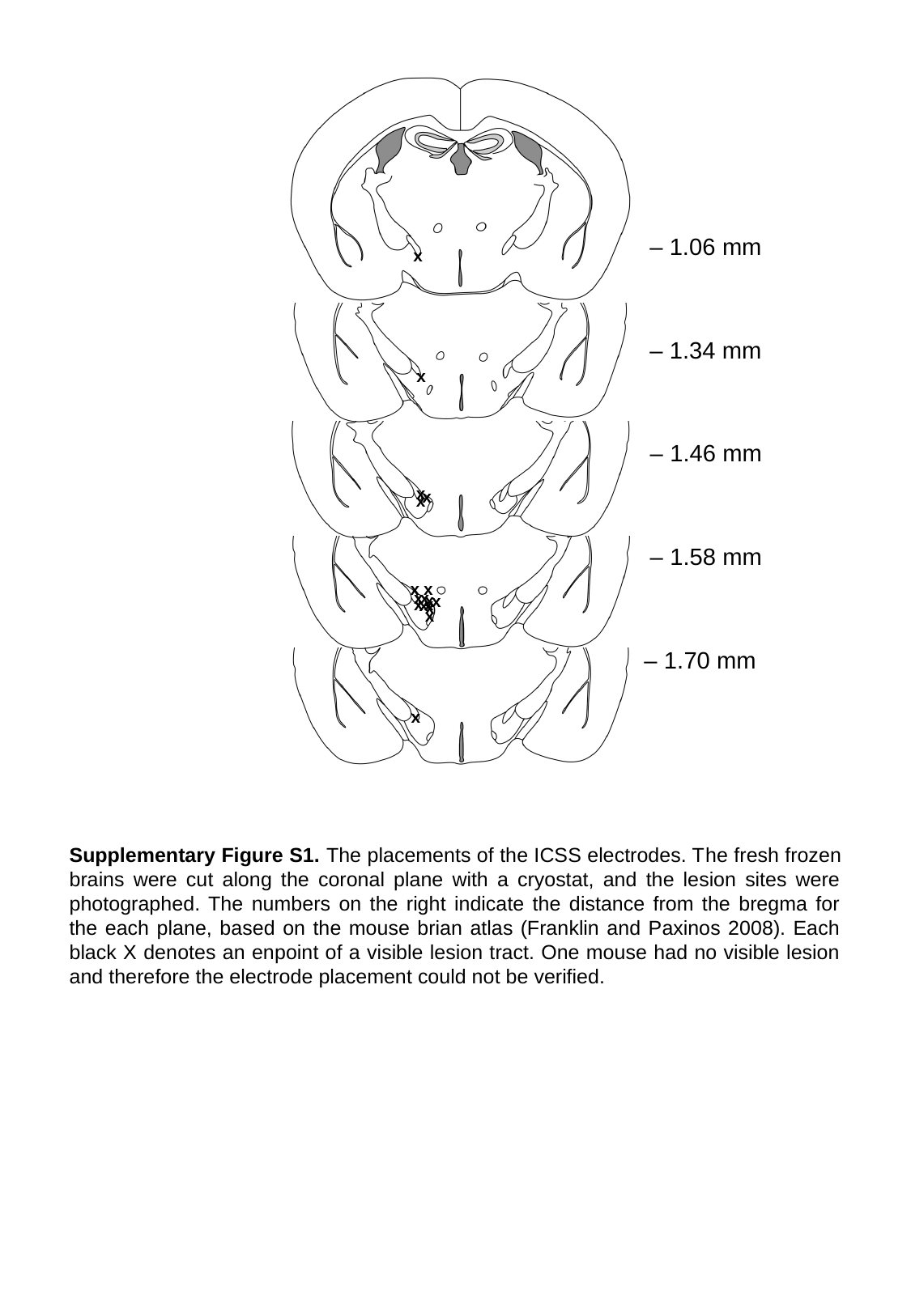

– 1.06 mm
x
– 1.34 mm
x
– 1.46 mm
x
x
x
– 1.58 mm
x
x
x
x
x
x
x
x
x
x
x
– 1.70 mm
x
Supplementary Figure S1. The placements of the ICSS electrodes. The fresh frozen brains were cut along the coronal plane with a cryostat, and the lesion sites were photographed. The numbers on the right indicate the distance from the bregma for the each plane, based on the mouse brian atlas (Franklin and Paxinos 2008). Each black X denotes an enpoint of a visible lesion tract. One mouse had no visible lesion and therefore the electrode placement could not be verified.
